# Comparison of conventional and CT-based planning for intracavitary brachytherapy for cervical cancer: target volume coverage and organs at risk doses

**DOI:** 10.1186/1756-9966-28-95

**Published:** 2009-07-01

**Authors:** Cem Onal, Gungor Arslan, Erkan Topkan, Berrin Pehlivan, Melek Yavuz, Ezgi Oymak, Aydin Yavuz

**Affiliations:** 1 Department of Radiation Oncology, Baskent University Medical Faculty, Adana, Turkey

## Abstract

**Background:**

To compare intracavitary brachytherapy (ICBT) planning methods for cervical cancer, based on either orthogonal radiographs (conventional plan) or CT sections (CT plan); the comparison focused on target volume coverage and dose volume analysis of organs at risk (OARs), by representing point doses defined by the International Commission on Radiation Units and Measurement (ICRU) and dose volume histograms (DVHs) from 3D planning.

**Methods:**

We analyzed the dosimetric data for 62 conventional and CT-based ICBT plans. The gross tumor volume (GTV), clinical target volume (CTV) and organs at risk (OAR)s were contoured on the CT-plan. Point A and ICRU 38 rectal and bladder points were defined on reconstructed CT images.

**Results:**

Patients were categorized on the basis of whether the >95% isodose line of the point-A prescription dose encompassed the CTV (group 1, n = 24) or not (group 2, n = 38). The mean GTV and CTV (8.1 cc and 20.6 cc) were smaller in group 1 than in group 2 (24.7 cc and 48.4 cc) (*P <*0.001). The mean percentage of GTV and CTV coverage with the 7 Gy isodose was 93.1% and 88.2% for all patients, and decreased with increasing tumor size and stage. The mean D2 and D5 rectum doses were 1.66 and 1.42 times higher than the corresponding ICRU point doses and the mean D2 and D5 bladder doses were 1.51 and 1.28 times higher. The differences between the ICRU dose and the D2 and D5 doses were significantly higher in group 2 than in group 1 for the bladder, but not for the rectum.

**Conclusion:**

The CT-plan is superior to the conventional plan in target volume coverage and appropriate evaluation of OARs, as the conventional plan overestimates tumor doses and underestimates OAR doses.

## Background

Intracavitary brachytherapy (ICBT) with external radiotherapy (ERT) is an essential component of cervical cancer management and has a high therapeutic index by delivering a high dose to the primary cervical lesion and lower doses to adjacent organs, resulting in increased local control and survival without increased in toxicity [1-4]. However the doses delivered to tumor and normal tissues from ICBT are difficult to quantify accurately in conventional brachytherapy (BRT) planning. To ensure consistency in the reporting of ICBT applications in cervical cancer, the International Commission on Radiation Units and Measurement (ICRU) recommended a number of parameters for doses and volumes to be considered. These include points A and B, representing the doses in the parametria and the pelvic wall, and the rectal and bladder points representing the organs at risk (OARs), respectively [5]. Physicians have used these reference point doses to report treatment intensity and to estimate the maximal dose to normal tissues, which can predict late complications. However, the conventional plan in treating cervical cancer may not correspond with the individual extent of the tumor. This may result in either undercoverage of the tumor or overdosage of the surrounding normal tissue.

A modern approach in treatment planning for cervical carcinoma is based on computed tomography (CT) sections and on a 3D dose distribution. This allows better assessment of dose distributions in different volumes, such as the gross tumor volume (GTV), clinical target volume (CTV), and OARs (rectum, bladder, and intestines). Ling et al. published the first report describing the volumetric dose distributions from ICBT [6]. In 2004, guidelines were published for proposing image-based BRT for cervical cancer [2]. However, the results of the first preliminary studies indicated that a great deal can be learned from volumetric analysis of ICBT dose distributions [7-9]. Furthermore, the actual doses delivered to the tumor, bladder, and rectum during ICBT do not correlate well with those estimated from ICRU reference-dose calculations, demonstrating that the point A dose in conventional plans overestimates the target volume dose coverage and underestimates the OAR doses determined by CT plans [10-12].

Although conventional treatment planning has generally yielded high tumor control rates, with a low frequency of major complications, a more accurate understanding of the radiation doses delivered during ICBT may lead to improved treatment outcomes. In an attempt to solve some of the problems that have limited the use of volumetric analysis of ICBT dose distributions and to achieve a better understanding of the treatments, we compared two treatment planning methods based on orthogonal radiographs (conventional plan) and CT sections (CT plan). The comparison was based on point doses defined by the ICRU and dose volume histograms (DVHs) from 3D planning.

## Methods

### Patient Characteristics

Between January 2008 and August 2008, 29 patients with uterine cervical cancer underwent radical concurrent chemoradiotherapy consisting of weekly cisplatin plus radiotherapy in the Department of Radiation Oncology at Baskent University in Adana, Turkey. Sixty-two BRT plans were evaluated. All patients were evaluated for staging with a thorough gynecological examination under anesthesia. Magnetic resonance imaging (MRI) was performed to assess local tumor extension and tumor size, and flouro-deoxyglucose (FDG) positron-emission tomography (PET-CT) was performed to assess lymph node and distant metastases. Baskent University's Institutional Review Board approved this study design.

### Treatment

The treatment consists of a combination of ERT with concurrent weekly 40 mg/m^2 ^cisplatin and high dose rate (HDR) BRT. All ERT was planned with a four-field box technique using a treatment planning system (Eclipse^®^, Varian Medical Systems, Palo Alto, CA, USA). A total of 50.4 Gy (1.8 Gy/fr, daily, Monday through Friday) was delivered using 18-MV photons. Brachytherapy was performed with a remote afterloading HDR unit with a radioactive Iridium-192 source (Varisource^®^, Varian Medical Systems). The ICBT procedure was initiated at the end of ERT. The median amount of time between the completion of ERT and the first BRT application was 2 days (range 1–5 days). The planned dose per fraction was 7 Gy prescribed to point A, given in 4 fractions, and the BRT was delivered twice weekly.

A CT compatible Fletcher-Suit applicators were used during ICBT application and consisted of uterine tandem with various angles (15°, 30°, 45°) and a pair of ovoids with various diameters (20, 25, 30 mm). Before each application, a urinary catheter was inserted and the catheter balloon inflated with contrast media (7 mL) to localize the bladder neck. Patients were not given specific instructions for rectal preparation, but they were encouraged to empty their bowels before a simulation procedure and before the next ICBT procedure. Appropriate anterior and posterior vaginal packing was used to fix the applicator position and to displace the bladder and rectum away from the vaginal applicators. After the intracavitary application, the applicator was fixed with a universal applicator clamping device (Varian^®^), which was underneath the patient. All patients underwent both conventional and 3D planning. To minimize patient movement during both the orthogonal films and CT scans, every attempt was made to keep the applicator in position and to complete the entire procedure within the shortest possible time. First, patients underwent orthogonal radiographic pelvic films for dose calculation. During conventional dose calculation, CT scans of the pelvis were performed with CT compatible applicators. Since the applicators are CT compatible, the shields were not used in order to overcome artifacts during CT scans.

### Conventional Planning

All patients had traditional radiography based treatment plans. The radiation source position, point A (left and right), point B (left and right), and ICRU reference bladder and rectal points were inserted in the planning system using orthogonal radiographic films obtained with metallic dummy markers inserted inside the applicator. The ICRU bladder reference point was identified using a Foley catheter, with the balloon filled with 7.0 mL of contrast material. The rectal point was defined as 5 mm behind the posterior vaginal wall (ICRU reference point), which could be visualized by radiopaque gauze used for the vaginal packing. The 7 Gy dose was optimized to Point A without making any modifications, such as weighting. During conventional planning, the doses to point A (right and left) point B (right and left), and the bladder and rectum were calculated. At the same time, volumes of the dose matrix receiving 50% (3.5 Gy), 100% (7 Gy), 150% (10.5 Gy), and 200% (14 Gy) of point A doses were computed.

### 3D CT-Planning

A CT scan with 2.5-mm slice thickness through the pelvis was performed for each HDR BRT in each patient with the CT compatible applicator in place. All CT slices were transferred, via a hospital network, to the treatment planning system (Brachyvision^® ^v 7.5, Varian Medical Systems) before a physician contoured the target volume and OARs on each slice of the CT scan. Dwell positions inside of the uterine tandem and ovoids were identified automatically from CT images using the planning system. The dose was optimized to target (CTV) minimum in order to receive at least prescribed 7 Gy. Delineation of the GTV was performed based on CT information at the time of the BRT and supported by clinical and radiographic findings, as recommended by 'Image-guided Brachytherapy Working Group'[2]. The Working Group proposes that the primary GTV be that defined through imaging plus any clinically visualized or palpable tumor extensions. This volume is meant to include the entire determinable tumor (the primary tumor in the cervix and its extensions to the parametria as determined by MRI plus the clinical examination). A safety margin for the GTV, which defines the CTV at the time of BRT, was calculated. In practice, the CTV covers the cervix plus the presumed tumor extension, reflecting macroscopic and microscopic residual disease at the time of BRT, which was proposed by the working group [2]. If the tumor extension at diagnosis was confined to the cervix proper, the CTV simply included the whole cervix. If there was parametrial infiltration, the depth of infiltration was estimated, and the safety margin was modified according to the parametrial infiltration depth. If the images showed a normal configuration of the corpus uteri, only the central part of the corpus was enclosed. If there was involvement of the fornices or the proximal vagina, these parts were included as well. Moreover, intra-observer variability was also assessed on 10 sample plans by a blind repetition of CTV contouring on randomly chosen CT scans. The average intraobserver variability was 0.5 mm and 0.7 mm for the cranial and caudal margins, respectively, with a maximum 0.9 mm intra-observer variation at the caudal limit of the CTV, which is in close proximity with literature findings [13,14].

Besides GTV, the external contour of the bladder, rectum, sigmoid colon, and small bowel in the pelvis were delineated on each CT slice by one physician. In this study, the rectum was delineated from the anal verge to the rectosigmoid junction, and the sigmoid colon was defined as the large bowel above the rectum to the level of the lumbosacral interspace. The bowel excluding the sigmoid colon and rectum in the pelvis was defined as small bowel.

After the ICRU reference points were identified on orthogonal films, they were transposed to CT images by co-registering the orthogonal films and digitally reconstructed radiographs (DRRs) obtained from CT scans. By this method, the point A dose simply transferred from the conventional plan to the conformal plan and then coverage compared. For co-registration, the software required the user to identify an origin, which was the external os in this study. As the external os was digitized on radiograph and CT, all reference points marked on orthogonal films were automatically transferred to CT films.

The DVHs of tumor volumes and OARs were created for each application. The volumes were calculated for the dose matrices receiving 50% (3.5 Gy), 100% (7 Gy), 150% (10.5 Gy), and 200% (14 Gy) of the point-A doses obtained from the conventional plan and the 3D CT plan. The extent of tumor coverage within the prescribed 7 Gy isodose volume obtained from orthogonal films and CT were compared. To compare the respective ICRU rectal and bladder point doses with the 3D volume dose, the minimum dose value in the 2.0-cc volume receiving the highest dose (D2) was determined from DVHs for bladder, rectum. The dose of a 5-cc volume (D5), which is defined as the minimum dose value in the 5.0-cc volume receiving the highest dose, was also calculated, because this volume was previously reported as the minimal volume required for fistula formation [7,8,15]. The Student's *t *test was performed for comparison of GTV, CTV, rectum, bladder, sigmoid colon, and small bowel volumes between groups. A comparison of the conventional plan and CT-plan was performed using the Wilcoxon signed-ranks test for all doses and volumes. *P *values less than 0.05 were considered statistically significant.

## Results

The mean age of the patients was 56 years (range, 26–77 years). Tumor stage was evaluated according to the International Federation of Gynecology and Obstetrics (FIGO) classification [16]. Two patients (7%) had Stage IB_2_, 3 (10%) had Stage IIA, 15 (52%) had Stage IIB, 1 (3%) had Stage IIIA, and 8 (28%) had Stage IIIB disease. Plans were categorized into group 1 (n = 24, 39%), where > 95% of the isodose line prescribed to point A in the conventional plan encompassed the CTV, and group 2 (n = 38, 61%), where < 95% of the prescribed point-A dose on the CT plan encompassed the CTV. The mean GTV and CTV in all patients were 14.1 cc (2.1–38.2 cc) and 36.3 cc (9.7–80.0 cc), respectively. The mean GTV, CTV, rectum, bladder, sigmoid, and bowel volumes according to groups are presented in Table 1. The mean GTV and CTV were smaller in group 1 than in group 2 (*P *< 0.001). The rectum, bladder, sigmoid colon, and small bowel volumes in all patients were 81.6 cc (37.5–177.6 cc), 60.3 cc (30.1–114.5 cc), 40.2 cc (10.8–62.8 cc), and 499.6 (158.1–973.3 cc), respectively. No significant differences were found between groups 1 and 2 in mean OAR volumes (Table 1).

**Table 1:** Mean values of GTV, CTV, and rectum, bladder, sigmoid colon, and small bowel volumes according to groups.

	**Group 1** **(cc **± **SD)**	**Group 2** **(cc **± **SD)**	***P***
**GTV**	8.1 ± 5.4	20.6 ± 12.3	< 0.001
**CTV**	24.7 ± 10.7	48.4 ± 20.8	< 0.001
**Rectum**	76.1 ± 37.7	82.3 ± 36.9	0.19
**Bladder**	57.8 ± 19.5	63.0 ± 19.9	0.24
**Sigmoid colon**	38.2 ± 15.2	40.5 ± 16.3	0.72
**Small bowel**	508.9 ± 193.6	488.9 ± 226.1	0.68

*Abbreviations: GTV = gross tumor volume, CTV = clinical target volume, Group 1 = CTV coverage > 95% isodose line prescribed to point A, Group 2 = CTV coverage < 95% isodose line prescribed to point A.

**Figure 1:**
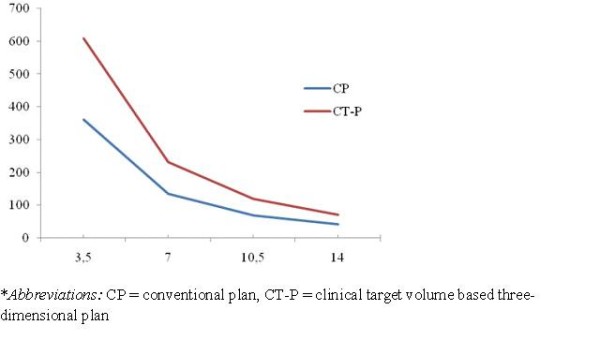
**Mean values of isodose volumes covering 50%, 100%, 150% and 200% of prescribed Point A 7 Gy dose**.

The volumes of the dose matrices for all patients receiving 50% (3.5 Gy), 100% (7 Gy), 150% (10.5 Gy), and 200% (14 Gy) of the point-A doses are shown in Figure 1. The mean isodose volumes at 3.5 and 7 Gy were significantly larger by CT-planning than by conventional planning (*P *< 0.001 and *P *= 0.01, respectively). However, no difference was found between conventional planning and CT-planning for the 10.5 and 14 Gy isodose volumes. Table 2 shows the volumes of the dose matrices receiving 50% (3.5 Gy), 100% (7 Gy), 150% (10.5 Gy), and 200% (14 Gy) of the point-A doses obtained from the conventional plan and 3D CT plan according to groups. With the conventional plan, the dose matrices receiving 50%, 100%, 150%, and 200% did not differ between groups. In both groups, the 7 Gy isodose volumes were significantly larger with the CT plan than with the conventional plan: 191.1 vs. 132.4 cc (*P *= 0.02), respectively, in group 1, and 266.8 vs. 137.4 cc (*P <*0.001), respectively, in group 2.

**Table 2:** The volumes of the dose matrix receiving 50% (3.5 Gy), 100% (7 Gy), 150% (10.5 Gy), and 200% (14 Gy) of point-A doses obtained from the conventional plan and the 3D CT plan according to groups.

	**Group 1 (cc)**	**Group 2 (cc)**	***P***
**Conventional plan**			
3.5 Gy	346.0 ± 81.3	375.4 ± 90.7	0.14
7 Gy	132.4 ± 31.5	137.4 ± 27.0	0.46
10.5 Gy	70.8 ± 18.6	69.5 ± 13.5	0.72
14 Gy	42.4 ± 12.8	41.7 ± 8.7	0.76
**3D CT plan**			
3.5 Gy	521.2 ± 127.3	685.7 ± 146.0	< 0.001
7 Gy	191.1 ± 46.5	266.8 ± 81.3	< 0.001
10.5 Gy	98.7 ± 26.5	135.1 ± 39.0	< 0.001
14 Gy	60.2 ± 18.4	78.9 ± 22.1	0.003

*Abbreviations: Group 1 = CTV coverage > 95% isodose line prescribed to point A, Group 2 = CTV coverage < 95% isodose line prescribed to point A.

### Target volume coverage

**Figure 2:**
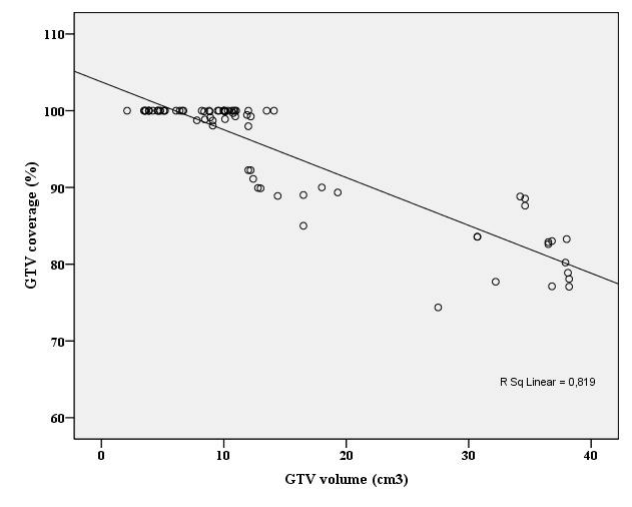
**Scatter-plot for gross tumor volume (GTV) vs. percentage of coverage of these volumes by the 7 Gy isodose**.

**Figure 3:**
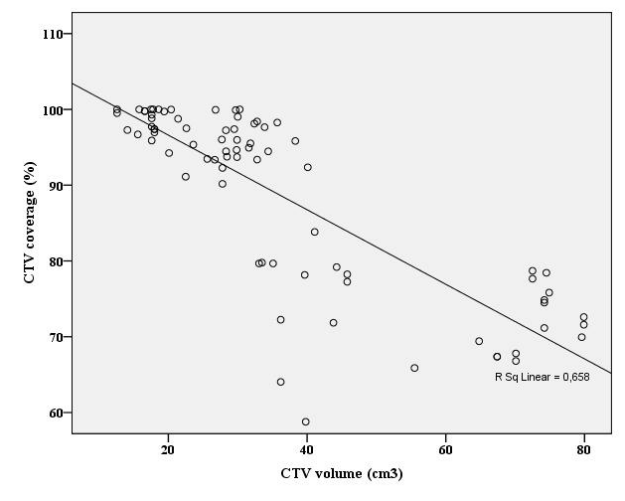
**Scatter-plot for clinical target volume (CTV) vs. percentage of coverage of these volumes by the 7 Gy isodose**.

When the dose was prescribed to point A, the mean percentage of GTV and CTV encompassed within the 7 Gy isodose level was 93.1% (74.4–100%) and 88.2% (58.8–100%) with CT plan respectively. The target volume coverage was inversely related to the volume of the target and the extension of tumor (Figures 2 and 3). In patients with larger tumors or tumors extending to the vagina or parametrium, the 7 Gy isodose line was more likely to not fully cover the GTV (Pearson correlation: -0.82, *P *< 0.001) and CTV (Pearson correlation: -0.80, *P *< 0.001) obtained from CT.

The mean GTV volumes according to stages were, 7.3 cc (3.5–11.9 cc) for IB_2_, 11.8 cc (5.1–34.6 cc) for IIA, 13.8 cc (6.1–36.5 cc) for IIB, 15.2 cc (7.8–34.2 cc) for IIIA, and 26.2 cc (7.6–48.2 cc) for IIIB diseases. Since GTVs were larger with the more advanced clinical stages, the GTV coverage with the 7 Gy isodose volumes decreased with increased tumor size and more advanced stage (Table 3). For stages IB_2_, IIA, IIB, IIIA, and IIIB, the mean CTV was 23.8 cc (12.6–33.9 cc), 31.0 cc (17.5–72.5 cc), 32.1 cc (18.1–74.2 cc), 37.3 cc (15.8–74.5 cc), and 56.0 cc (22.6–89.9 cc), respectively. Similarly the CTV coverage with the 7 Gy isodose volumes diminished with more advanced stage (Table 3).

**Table 3:** Mean GTV and CTV and coverage of these volumes by the 7 Gy isodose according to clinical stage.

**Stage**	**GTV volume (cc)**	**GTV coverage (%)**	**CTV volume (cc)**	**CTV coverage (%)**
**IB2**	7.3	99.9	23.8	98.9
**IIA**	11.8	97.1	31.0	94.4
**IIB**	13.8	94.4	32.1	89.9
**IIIA**	15.2	93.5	37.3	90.6
**IIIB**	26.2	86.5	56.0	77.9

*Abbreviations: GTV = gross tumor volume, CTV = clinical target volume.

### Rectum doses

We compared the ICRU rectum and bladder point doses, based on the conventional plan, with the D2 and D5 of the rectum and bladder, based on the CT-plan. The mean ICRU rectal dose obtained from the conventional plan for all patients was 5.0 Gy (2.2–10.7 Gy), and the mean D2 and D5 of the rectum obtained from the 3D plan were 8.3 Gy (5.1–12.3 Gy) and 7.1 Gy (4.5–11.1 Gy), respectively. The mean D2 and D5 of the rectum were 1.66 and 1.42 times higher than the mean ICRU rectum dose. The paired difference between ICRU rectum point dose and D2 (*P *< 0.001), and D5 (*P *< 0.001) demonstrated a significant difference for all patients (Table 4).

**Table 4:** Mean values of organs at risk using the ICRU reference point doses with the conventional planning method and the D2 and D5 values using the 3D CT planning method.

	**Group 1** **Gy (%)**	**Group 2** **Gy (%)**	***P***
**ICRU**			
**Rectum**	6.2 (89.0)	5.9 (84.7)	0.34
**Bladder**	5.2 (74.2)	4.9 (69.9)	0.51
**D2**			
**Rectum**	8.1 (116.0)	8.5 (120.8)	0.46
**Bladder**	8.6 (122.3)	9.7 (138.8)	0.03
**Sigmoid**	5.9 (84.4)	7.1 (100.5)	0.009
**Bowel**	6.3 (90.1)	7.2 (103.5)	0.07
**D5**			
**Rectum**	7.0 (100.0)	7.2 (103.5)	0.43
**Bladder**	7.3 (104.0)	8.2 (117.4)	0.03
**Sigmoid**	4.6 (65.4)	5.5 (78.2)	0.02
**Bowel**	5.3 (75.6)	5.8 (83.9)	0.2

*Abbreviations: Group 1 = CTV coverage > 95% isodose line prescribed to Point A, Group 2 = CTV coverage < 95% isodose line prescribed to Point A.

The mean rectum ICRU point doses and D2 and D5 values did not differ significantly between groups 1 and 2 (Table 4). However, within each group, the differences between the ICRU rectum dose and D2, and between the ICRU rectum dose and D5 were significant. The differences in rectum doses between ICRU and D2, and ICRU and D5 were greater in group 2 than in group 1, but this did not reach statistical significance (Table 5).

**Table 5:** Differences between ICRU rectum and bladder doses from orthogonal films and D2, and D5, of the rectum and bladder obtained from CT scans.

**Variable**	**Mean difference (Gy)**			
	All patients	Group 1	Group 2	*P *(1 vs.2)
				
**Rectum**				
ICRU-D2	-3.3	-2.9	-3.6	0.24
ICRU-D5	-2.1	-1.8	-2.4	0.26
Bladder				
ICRU-D2	-3.1	-2.3	-3.8	0.01
ICRU-D5	-1.7	-1.1	-2.3	0.01

*Abbreviations: Group 1 = CTV coverage > 95% isodose line prescribed to Point A, Group 2 = CTV coverage < 95% isodose line prescribed to Point A. D2 = the minimum dose value in the 2.0-cc volume receiving the highest dose, D5 = the minimum dose value in the 5.0-cc volume receiving the highest dose.

### Bladder doses

The mean ICRU bladder dose and D2 and D5 of the bladder for all patients were 6.1 Gy (2.9–8.7 Gy), 9.2 Gy (7.6–12.9 Gy), and 7.2 Gy (3.4–10.9 Gy), respectively. The mean D2 and D5 of the bladder were 1.51 and 1.28 times higher than the mean ICRU bladder dose (6.1 Gy and 5.6 Gy). The differences of means between the ICRU bladder dose points from the conventional plan and the D2 (*p *< 0.001) and D5 (*p *< 0.001) of the bladder from the CT plan were statistically significant.

The mean ICRU bladder doses did not differ between groups 1 and 2. However, D2 and D5 values were significantly higher in group 2 than in group 1 (Table 5). Likewise, there were significant differences between ICRU bladder and D2 values (*p *< 0.001) and D5 values (*p *< 0.001) for groups 1 and 2. The difference in the ICRU bladder point dose and D2, and the ICRU bladder point dose and D5 was significantly higher in group 2 than in group 1 (Table 5).

### Comparison of sigmoid colon and small bowel doses

The mean sigmoid colon and small bowel doses for all patients were 6.5 Gy (2.6–11.2 Gy) and 5.1 Gy (2.1–9.8 Gy), respectively, for D2; and 6.8 Gy (2.0–11.5 Gy) and 5.6 Gy (1.8–9.7 Gy), respectively, for D5. The D2 and D5 values for sigmoid colon were significantly higher in group 2 than in group 1 (up to 15%) (Table 4). Although the D2 and D5 values for the small bowel were also higher in group 2 than in group 1, the difference did not reach statistical significance.

## Discussion

In the current study, we assessed the conventional BRT plan based on ICRU reference points and the CT-based BRT plan in patients with cervical cancer. We clearly demonstrated that tumor volume coverage was inadequate in the conventional plan compared to the CT-plan, and was inversely related with the volume of the target and the extension of tumor. With the conventional plan, the ICRU rectum and bladder point doses underestimated the actual rectum and bladder doses obtained from the CT-plan. Additionally, we demonstrated that more precise analysis of the dose received by certain volume of OARs can be accomplished by utilizing the DVHs on CT-plans, which may be of critical importance in regard to normal tissue tolerance limits.

After publication of ICRU 38 report, ICRU reference points for tumors, and reference dose points for bladder and rectum were used for defining the doses in conventional plans. But calculation of doses with these fixed reference points relative to applicators has certain limitations. The conventional plan with the point-A dose calculation relies on reference points on orthogonal films, not tumor volumes defined on CT, which may cause underestimation of tumor doses. Likewise, the calculation of rectum and bladder doses made with ICRU reference points, not with rectum and bladder volumes, may not reflect the actual organ doses. In addition, sigmoid colon and small bowel in the pelvis may be in close proximity to the BRT sources during application, and the doses to these organs should also be assessed. Since the ICRU did not define standard points for the sigmoid colon and small bowel, it is not possible to evaluate doses to these organs with conventional plans. To overcome such problems, CT-guided 3D BRT treatment planning has been used successfully for customizing the dose distribution according to tumor extent and providing detailed dose-volume information on the target volumes and surrounding tissues [12,17-21].

Some investigators have reported that the point A-dose in the conventional plan overestimates the target volume dose coverage [10-12]. In addition, more advanced tumor stages and larger target volumes receive less coverage with the prescribed dose, which may result in poor local control [12,22]. Datta et al. demonstrated that the percentage of tumor encompassed within the point-A dose envelope ranged from 60.8% to 100%, and this percentage depended on the tumor volume at the time of ICBT [18]. In the current study, we demonstrated that the mean percentage of GTV and CTV encompassed within the point-A 7 Gy isodose level was 93.1% (74.4%–100%) and 88.2% (58.8%–100%), respectively. Inadequate tumor coverage could significantly influence the treatment outcome in patients, especially in those who have partial regression of tumors with gross residual tumor after ERT. Thus, tumors with larger volumes at ICBT were more likely to have portions outside the 7 Gy prescribed isodose line (Figures 2 and 3). Initially, Kim et al. demonstrated that the CT-plan would be beneficial in patients with large CTVs, which could not be fully encompassed by the 100% isodose line [12]. In the current study, the GTV and CTV were larger in group 2 than in group 1; therefore, the CT-plan would be most beneficial in group 2. Although the isodose matrix volumes did not differ between the two groups with the conventional plan, these volumes were higher in group 2 with the CT-plan (Table 2), which may cause a significant incremental dose to the neighboring tissues, mainly the bladder and sigmoid colon (Table 3).

Although tumor shrinkage before BRT applications may take place after ERT, the initial tumor stage, which reflects the tumor extension, may negatively impact tumor coverage [1,22,23]. Kim et al. demonstrated that GTV but not CTV increased with advanced stages [23]. They also found that the percentages of the GTV encompassed by the 6 Gy isodose line were 98.5%, 89.5%, 79.5%, and 59.5% for stages IB_1_, IB_2_, IIB, and IIIB, respectively. In our study, the GTV and CTV appeared to increase with more advanced clinical stages. Meanwhile, tumor coverage within the 7 Gy isodose line diminished with more advanced clinical stages (Table 3). Therefore, a higher stage of tumor received less coverage by the prescribed point-A dose because of extension to the parametria and/or vagina.

For evaluating the maximum doses to OARs, the dose to a clinically significant volume is used; that clinically significant volume can be defined as the volume exposed to a minimum dose in the part of the OAR that receives the highest dose. The size of this volume can be absolute (e.g., 1, 2, 5, or 10 cc) or relative (e.g., 1%, 2%, 5%, or 10% of the contoured OAR). Several investigators have compared the dose volume based on either the exterior organ contour or only the organ wall, for the bladder and rectum [8,24,25]. To evaluate organ wall dose correctly, the volume of 2.0 cc is considered, because the D2 computed for the external contour are almost the same as the D2 to the organ wall. Also, this 2.0 cc volume of tissue in the highest dose region is probably more clinically relevant. Although the difference between the DVHs increases greatly for volumes larger than 2.0 cc, we also chose the dose of a 5-cc volume (D5), because this volume was previously reported as the minimal volume required for fistula formation [7,8].

The rectum and bladder doses were found to be greater than the corresponding ICRU reference doses [7,8,12,18,26]. In these other studies, the true bladder and rectum doses were 1.5–2.5 times greater than the corresponding ICRU reference point doses. Pellioski et al. compared the minimal doses delivered to 2 cc of the bladder and rectum (D_BV2 _and D_RV2_) and found that ICRU bladder reference point dose was significantly lower than the D_BV2_, but the ICRU rectum reference point dose was not significantly different from the D_RV2 _[26]. Our study indicated that the maximum rectum and bladder D2 values were 1.66 and 1.51 times greater than the ICRU reference rectum and bladder doses, respectively. We also found that the maximum rectum and bladder D5 values were 1.42 and 1.28 times greater than the ICRU reference rectum and bladder doses in CT plan. When we evaluated the difference between the ICRU rectum and bladder doses and corresponding D2 and D5 values, the differences between the ICRU bladder point dose and D2 and D5 bladder doses were significantly higher in group 2 than in group 1; however the difference in rectal doses did not differ significantly (Table 5).

Since the sigmoid colon and small bowel in the pelvis are close to the radiation source during ICBT, doses received by these organs should also be assessed. The ICRU defined the reference points for bladder and rectum, the initial dose calculations for these organs were performed during the conventional plan. In addition, the doses to the sigmoid colon and small bowel can be evaluated with the CT-plan using DVHs. Al-Booz et al. pointed out that the sigmoid colon received doses in excess of 70% of the intended point-A dose during BRT [27]. Kim et al. demonstrated that that the sigmoid colon received the highest mean D2 when compared to the rectum and small bowel [28]. Their study revealed that with the prescribed dose of 600 cGy, the sigmoid colon received the highest mean D2 (408 cGy) followed by the small bowel (379 cGy), and rectum (373 cGy). In our study, we clearly demonstrated that the small bowel D2 was higher than the sigmoid colon D2 (6.8 Gy and 6.5 Gy, respectively). We also found that the sigmoid colon D2 and D5 values were significantly higher with larger CTVs (Table 4). The small bowel D2 values were higher in group 2 than in group 1, and this difference was almost statistically significant (*P *= 0.07).

The results of our study demonstrate that CT-guided BRT planning is superior to conventional point A planning in terms of both conformity of target coverage and evaluation of OARs, including the sigmoid colon, bowel, bladder, and rectum. Although this superiority was clear for small CTVs, for large CTVs both the conventional and CTV plans had the drawbacks of inadequate target coverage and/or excessive radiation doses to normal organs. To ascertain the potential benefit of treatment outcomes, such as tumor control probability and morbidity, ICR with image-guided 3D planning will be pursued and correlated with the dose-volume parameters.

## Competing interests

We have no personal or financial conflict of interest and have not entered into any agreement that could interfere with our access to the data on the research, or upon our ability to analyze the data independently, to prepare manuscripts, and to publish them.

## Authors' contributions

All authors read and approved the final manuscript. CO prepared the design of the manuscript and made the contouring of the target volume and organs at risk; ET and EO collected the samples; AY gave advise on the work and MY helped in the interpretation of the data; GA made the treatment planning; CO wrote the paper together with BP.
